# Obituaries of the Late Drs. Carothers and Dr. Tyler

**Published:** 1890-08

**Authors:** 


### Society Notes.

### OBITUARIES.

[At the recent meeting of the West Texas Medical Association at San Antonio, the following papers were read and referred to the JOURNAL for publication.—ED.]

### THE LATE A. E. CAROTHERS, M. D.

A. E. Carothers was born in Carlisle, Penn., of a family of Scottish decent, in modest circumstances, but of most excellent record; his grandfather having been at Valley Forge with Washington, and two of his uncles having fallen in the war of 1812. A maternal uncle, Peter Kerr, was well known in the early annals of Texas, and survived her independence many years. Young Carothers received a good English education in the high school of his native place which enabled him to eke out his scanty means in studying for the profession he had chosen. During his boyhood he spent some time in Texas with his Uncle Peter, and accompanied the expedition of Captain, afterwards Major General Pope, in Texas and New Mexico. He returned to Pennsylvania, and early in the war was appointed, although yet an undergraduate, Assistant Surgeon of a Pennsylvania regiment. At this time party politics ran high, embittered as they were by the gigantic struggle between the North and South. Carothers was an ardent Democrat, and chastised an officer, who in his presence slandered the Democratic candidate for the gubernatorial chair of the State of Pennsylvania. For this he was dropped from the service, and most young men in his place, would have seen no other resource than to shoulder a musket. However, there was in the lad that which would not yield to the "slings and arrows of outrageous fortune," and this trait in his character we have seen well exemplified in later years. He returned to Philadelphia, graduated at the University of Pennsylvania, presented himself for examination for appointment to the Medical Staff Corps, passed, was appointed and married all within six months.

He must have stood well with the examining board, for he was immediately on his appointment assigned to duty in the hospital for wounded officers in Washington City. In that excellent school he studiously and industriously prepared himself for future excellence in his profession. At the close of the war he was ordered to Texas. Dr. Carothers arrived in San Antonio as Assistant Surgeon, Medical Staff Corps, serving with the troops which occupied the city after the close of the war in 1865, under command of General Merritt, and was shortly afterward mustered out of the service. He decided on settling, here and in 1866 was appointed on the Board of Health, and his activity and intelligent energy contributed greatly to check the epidemic of cholera which prevailed in September and October of that year. He was fast building up a good practice when in 1867 his health failed and repeated hemorrhages from the lungs, and hectic fever marked the gravity of the situation. By the advice of friends, and in full accordance with his own views he went to Saltillo, Mexico, where his health was fully re-established, and he was equal to the fatigues of a full practice. His reputation in general surgery, and more especially as a lithotomist, was wide spread on the border. He made thirty-four lithotomies with two deaths, an excellent record, many of his cases being of great interest. From one patient he removed thirty-eight calculi, of the size of a filbert, and as uniform as if they had been cast in the same mould. In his first lithotomy, done in San Antonio in 1867, he made the semi-lunar incision, adding a vertical one to make room for a calculus of greater dimensions than he had calculated on. In his subsequent operations he employed the time-honored lateral operation. In this connection I may mention a fact which deserves to be known, that in Austin, Texas, in 1867, he made a perineal section for severe cystitis of long standing, and with perfect results; this method is, as we all know, to-day universally adopted. In 1870 he made an intermediary hip joint amputation for gun-shot wound, the subject being a Mexican soldier, the result of which is best shown by the photogragh taken eighteen years after the operation. This case was published at the time in Hay's Philadelphia Journal of the Medical Sciences. In 1879 he made the first tracheloraphy done in Texas. In the same year he for the first time in surgery placed a drainage tube in the cerebral tissues, the patient making a perfect recovery. As a New York surgeon a few years ago published a case which he claimed to be the first of the kind, I may state that in the presence of the late Dr. Gaenslen, of Dr. F. Herff and of the writer, Dr. Carothers after removing five crown heads of the trephine and removing a large splinter of bone which lay on the dura mater, finding that there was no dimunition of the profound. coma which had existed for three days, diagnosed intra-meningeal pressure, and having located an abscess in the cortical tissue, proposed to open it by incision. One of the gentlemen assisting in the operation, suggested the preliminary use of the needle syringe. This was done and the presence of pus demonstrated. An incision was made, and about two drams of pus evacuated, a drainage tube inserted, the coma passing off with the removal of the pressure. The recovery was uninterrupted but for a hernia cerebri, for which the scalp flaps were securely sutured and supported by a bandage to the complete recovery of this troublesome complication.

During his stay in Mexico Dr. Carothers removed for necrocis both the superior maxillar, molar bones, and pterygoid process of the sphenoid of which I present photographs.

Dr. Carothers was gifted by nature with many qualifications which admirably fitted him for the practice of surgery, he was cool and self-reliant, possessing the assurance born of a clear knowledge of what was before him; a fair anatomist, he was prepared for any emergency; quick in perception, prompt in action, his clear intelligence was never at fault, and the writer gratefully acknowledges the able assistance, and the still more valuable counsel of our deceased colleague in many of the most important operations in surgery. It is a matter of lasting regret to his friends, and a great loss to the profession, that he abandoned medicine for other pursuits. These presented every promise of success, but unforseen disaster came upon him, and at the very moment when a new vista of most promising operations opened to him, he imprudently exposed himself in the City of Mexico after an attack of influenza, acute pneumonia set in, and after three days sickness, two of which were passed in total insensibility, he paid the last tribute which man is called upon to offer. Peace be with him. He will long hold a large place in the memory and regrets of his friends, regrets to be in a few short weeks enhanced by the tragic fate of the son on whose career in life he had built so many hopes. Of happy temper and cheerful disposition, Dr. Carothers was a most genial companion, and made hosts of friends wherever he went. He was independent in thought, and framed his convictions for himself. In his professional capacity he never "bent the pregnant hinges of the knee that thrift might follow fawning," and he ever maintained the honor and dignity of the noble profession which he honored and illustrated by his work. Dr. Carothers was one of the founders of this Association, member of the Texas State Medical Association, and of the Philadelphia Academy of Sciences.

GEO. CUPPLES, M. D.

---

### IN MEMORY OF DR. E. J. CAROTHERS.

Again it becomes our melancholy duty to notice the death of another member of this Association, Dr. Edward J. Carothers, which occured in Saltillo, Mexico, on the 9th day of April last, by the accidental discharge of a pistol in his own hands. Only a few days before his tragic death he was in this city amongst his friends, in splendid health, full of hope, and with the promise of long life; to-day his body fills its place in the dark chambers of the dead, and his spirit has returned to the God who gave it.

The subject of this memorial was born in Carlisle, Penn., in 1863. He graduated in medicine at the University of Pennsylvania, in 1885, and after spending about two years in this city, he removed to Saltillo, Mexico, where the early years of his boyhood were spent, and where his father had made such a brilliant reputation as a surgeon. He immediately started into a good practice, and had before him a good prospect for success and future usefulness, when he met his tragic end. Now the West Texas Medical Association, in its regular quarterly meeting, in the time set apart as the memorial hour, do make these resolutions:

WHEREAS, The members of the West Texas Medical Association have heard with profound sorrow of the tragic death and untimely end of our Brother, Dr. E. J. Carothers, who was killed by the accidental discharge of a pistol on April 9th,

*Resolved*, That the West Texas Medical Association has lost a worthy member, and the profession a zealous and rising young physician, That we tender his bereaved family our heart-felt sympathy in their great affliction. That these memorial proceedings be spread on the minutes of the Association. That the Secretary be requested to furnish the family with a copy of these resolutions.

GEO. CUPPLES, M. D.,
T. R. CHEW, M. D.,
C. E. R. KING, M. D.
Committee.

THE LATE DR. E. H. TYLER, OF SAN ANTONIO.

The subject of this memorial, Dr. E. H. Tyler, was born in Owensboro, Ky., in 1851. After spending his boyhood and receiving his education in his native town, he begun the study of medicine in Bellevue Hospital Medical College, New York, and was graduated from that institution in 1878. Dr. Tyler practiced medicine a short while in St. Louis, Mo., and leaving there came to San Antonio in 1882, where he successfully engaged in the practice of his chosen profession, winning the affection and esteem of all who ceme in contact with him, and setting an example in his every day walk of life eminently worthy emulation by his surviving friends. He was an earnest and sincere Christian, and member of the First Baptist Church of this city. He was also a prominent member of the Y. M. C. A., and at one time one of the directors of the same. He served for two years —'84 and '85—as Secretary of the West Texas Medical Association, rendering his services with fidelity, punctuality and efficiency. He remained with us in San Antonio about six years, leaving in '88 to go to Buffalo Lithia Springs, Va., in search of relief from the use of the waters for diabetis insipidus, with which disease he had been suffering some time. Finding the water of not much benefit, after remaining there for about a month he left for New York, where he placed himself under the care of Dr. Janeway for treatment. Dr. Tyler remained in New York for about three months, and receiving no permanent benefit, he returned home to Owensboro, Ky., where, being unable to engage in the practice of medicine, he took a position in an insurance office. He died at his home in Owensboro, early in April, 1890.

While we feel grieved and regret the loss of our friend, knowing it to be inevitable, we at the same time must feel grateful to know that he whose life was devoted to the relief of suffering humanity and the service of God, has received the reward of the faithful, and is happy.

GEO. CUPPLES, M. D.,
T. R. CHEW, M. D.,
C. E. R. KING, M. D.,
Committee.

